# From U. Kreit, D.D.S.

**Published:** 1882-12

**Authors:** U. Kreit

**Affiliations:** Detroit


### CORRESPONDENCE.

DETROIT, NOV. 9th, 1882.

EDITOR OF REGISTER,

Dear Sir;—Herewith I give you the historyof a partial anæthesia of the facial and tri-facial nerve. About fourteen days ago a strong, robust, healthy-looking German man, thirty-three years of age, came into my office to consult me in regard to his facial trouble.

The history of his case is as follows: In the middle of September last he was traveling from East Saginaw to the city of Detroit on the railroad. At once, he said, he had a curious feeling on the right side of his face, and was not able to eject the saliva from his mouth, or shut his right eye. The sense of taste was also impaired, or different from before. When he came home he went to bed, thought everything would be well in the morning. The next morning he found the right side of the face red and swollen, his nose and mouth was drawn towards the left side of the face; his eyesight was affected, but there was no disturbance of the masticating muscles, and no pain. He went to a prominent physician of Detroit for consultation; he examined him, and pronounced the case Erysipelas, and prescribed for it. After a few days treatment he changed his diagnosis to facial paralysis. After a weeks treatment without any effect, the patient left the physician and went to another, with the same result. After three weeks treatment and consultation with six different physicians, and no change in his case, he came and asked me for advice. He asked me if the teeth could produce such trouble; I answered affirmatively; he requested me to examine his mouth, which I did, and found the following condition; the gums of the superior maxillary in a congested, inflamed and scorbutic state, and more on the affected side; there was a large accumulation of tartar on the cuspid and first bicuspid on the same side; the first bicuspid was somewhat decayed but no nerve exposed. I found also the dead root of the second bicuspid; the mouth on that side was dry, no discharge of saliva through the duct of steno. I made pressure to the apex of the root of the above named teeth and the patient said he felt pain up to his eye, in the region where the anterior central nerve runs down; he had also pain in the parotid gland; without pressure there was no feeling. I told him that I believed the teeth and parotid gland were the cause of his trouble; I recommended him to have the tartar removed and the tooth cleaned and the dead tooth extracted. As he was at that time under the treatment of a physician, I told him to go and say to him what I have recommended. After a short time he came back and said that the physician acquiesced; I operated at once, cleaned and removed the tartar, extracted the dead root and lanced his gums freely; I gave him the following as a mouth wash:

| | | |
|---|---|---|
| ℞. | Potassia chlorate, | ʒiii |
| | Aqua, | ℥ii |

Dissolve; add half pint of water, and use *ad libitum.* He left my office to report again in two days. When he come back I examined his oral cavity and found his gums much improved. He said he felt a great deal better in his mouth, but the swelling of his face was the same as before. I thought the swelling came from the congested and inflamed parotid gland and advised him to have four leeches applied to the affected parts. I put on four leeches, three to the gland and one to the oribital region, under the oribital cavity near to the nose, where he felt the pain by pressure. I told him to let it bleed from two to three hours, and report again; next day he came back the swelling was almost gone; to relieve the swelling I ordered the application of warm or cold poultice as he could bear it. Through the use of the leeches and poultices the swelling subsided, the nose and mouth returned to the normal state, but he was not able yet to shut his eye. As there was no further pain by pressure I recommended him to use the electric battery. He is improving daily, the parotid gland is active, and secretion is established through stenos duct; his health and appetite otherwise are good, the sight is improved, but the muscles orbicularus palpebrarum and tensor tarsi have not returned to the full activity yet. What was the cause of this disturbance? Was it from the draft in the car, on the parotid gland that produced congestion, and through the congestion pressure on the facial nerve branches, or was it from the irritation of the dead root and by accumulation of tartar on the dental nerve?

Since I have written this the patient called again; he complains of pain in the lower jaw. I examined him again found the gum of the lower jaw more inflamed than at first, (the gums of the upper jaw still better). I lanced the gum again, ordered the same mouth wash as before, and as he is careless in cleaning his teeth, I insisted that he use the tooth brush, also the further use of the battery. What else can be done? When the final recovery occurs I will report it to you.

Yours truly

U. Kreit, D. D. S.

